# Clinical Implications of the Lung Ultrasound Score in Patients after Cardiopulmonary Resuscitation

**DOI:** 10.1155/2023/4951950

**Published:** 2023-12-26

**Authors:** Yi-Ling Zhang, Zhou Yang, Jie Cao, Yu-Long Bai, Chun-Yun Fang, Wei Wang

**Affiliations:** Department of Emergency, The First Affiliated Hospital of Guangxi Medical University, Nanning, China

## Abstract

**Background:**

Lung ultrasound score (LUS) is a clinical index used to measure lung injury, but its clinical value in patients after cardiopulmonary resuscitation (CPR) remains relatively unknown. The purpose of this study was to investigate the clinical value of LUS in patients after CPR.

**Methods:**

This retrospective study included a total of 34 patients older than 18 years with a nontraumatic cause of in-hospital cardiac arrest, who received standard resuscitation and achieved return of spontaneous circulation (ROSC). All patients underwent bedside lung ultrasound examination within half an hour once ROSC was achieved, and LUSs were calculated. The study included patient death as the endpoint event.

**Results:**

Compared with the group with lower LUSs, the patients with higher LUSs had a lower oxygenation index, longer duration of CPR, and lower 72 h survival rate. The initial LUS had good clinical value in predicting the secondary outcomes of CPR (adjusted odds ratio (aOR): 1.353, 95% confidence interval (CI): 1.018–1.797, and *P* = 0.037) and 72 h survival rate of patients who underwent CPR (aOR: 1.145, 95% CI: 1.014–1.294, and *P* = 0.029).

**Conclusions:**

LUS was shown to be helpful and had a prognostic value in patients after CPR.

## 1. Introduction

Patients who experience cardiac arrest are greatly benefitted by the crucial skill of cardiopulmonary resuscitation (CPR) for return of spontaneous circulation (ROSC). Current CPR guidelines recommend high-quality chest compressions, which includes chest compressions with a depth of at least 5 cm [1–3]. Current research indicates that standard CPR compressions do improve the survival rate but postcardiac arrest syndrome is a multiorgan dysfunction mainly caused by heart and brain damage due to systemic ischemia-reperfusion injury after resuscitation, which is still the main cause of death of patients [4], and inevitably increases the risk of lung injuries related to CPR [5]. At present, the research of organ injury after resuscitation mainly focuses on the heart and the brain, and the lung as an important organ is relatively less studied. Severe lung injuries may lead to decreased oxygenation and ventilation and secondary adverse events like hypoxic ischemic encephalopathy and affect patient's outcomes. Thus, early imaging is required to help appropriately diagnose and manage resuscitated patients.

Most of the traditional lung injury evaluation indicators are invasive, such as PaO_2_/FiO_2_, which need to be extracted from arterial blood for blood gas analysis. Studies have shown that changes in PaO_2_/FiO_2_ do not lead to early identification of lung injury [6]. The early diagnostic value of bedside chest radiographs is limited and radiative [7]. In recent years, with the advent of rapid examination, thinner image slices, and multifaceted reconstitution capacity, multidetector computed tomography (CT) has been increasingly used in patients with cardiopulmonary arrest. A chest CT scan is the best method to evaluate lung injuries associated with CPR because it can provide a complete image of the chest structure. However, chest CT is not suitable for patients who are unstable due to the need for patient transport [8], which puts such patients with unstable condition at a high risk. Pulse indicator continuous cardiac output (PICCO) allows for bedside, continuous, reliable, and precise monitoring of cardiopulmonary conditions in resuscitated patients [9], but it is invasive and might lead to a number of complications. Lung ultrasound has the advantages of noninvasive, nonradioactive, simple, rapid, and reproducible and has been widely used in the evaluation of acute and critical diseases assessment [10], including pneumothorax, pleural effusion, and pulmonary edema. However, there are few studies on its application value in the assessment of lung injury after resuscitation [11].

Currently, ultrasonography is the only technique that enables procurement of images at the patient's bedside. This permits timely identification of organ complications, such as that of the lungs, decreases the need to move unstable patients, and reduces the risk of contagiousness. Lung ultrasound not only has the advantages of simplicity, speed, safety, no radiation, and high repeatability but also clinicians can quickly master this technology through short-term learning and training [12]. According to recent research, the lung ultrasound score (LUS) is an independent predictor of worse prognosis in patients with lung lesions [13, 14]. However, no studies have reported the clinical value of LUS in patients with CPR-related pulmonary injuries. Our study aims to evaluate if the initial LUS after ROSC was associated with worse outcomes in patients with cardiac arrest.

## 2. Materials and Methods

### 2.1. Patients and Methods

The was a retrospective study conducted in the emergency intensive care unit of the First Affiliated Hospital of GuangXi Medical University for over 29 months (July 2020 through May 2022). Patients over the age of 18 with nontraumatic cardiac arrest and who achieved ROSC were enrolled in this research. This study was approved by the Ethics Committee of the First Affiliated Hospital of GuangXi Medical University. We reviewed the patients' electronic medical records (EMRs) to assess the cause of cardiac arrest, any underlying disease, CPR duration, adverse events secondary to CPR, successful attainment of ROSC or not, and the 72 h survival rate for each patient. In our study, death was the primary outcome, while refractory hypotension, neurological dysfunction, and malignant arrhythmia after CPR were considered as secondary outcomes. ROSC refers to the restoration of spontaneous, sinus, or supracentricular heart rhythm and the systolic blood pressure was ≥50 mmHg (1 mmHg = 0.133 kPa), and the above indices were maintained for ≥20 min [15]. ILCOR statements recommend burst suppression on EEG at ≥24 h from ROSC combined with other indicators to predict poor outcome in adult patients who are comatose [16]. According to the professional quality control index of Emergency Medicine of China National Health Commission, ROSC success refers to the recovery of spontaneous breathing and circulation for more than 24 hours after cardiopulmonary resuscitation. Therefore, we took 24 hours after ROSC as the time point to determine whether resuscitation was successful and patients who died within 24 hours after ROSC were considered to have failed resuscitation.

Based on the results of clinical, imaging, and laboratory examinations, the causes of cardiac arrest were determined and categorized into cardiac, respiratory, cerebral, septic, metabolic causes, and drug overdose. Cardiac causes included acute coronary syndrome and heart failure; respiratory diseases included asphyxia, severe asthma, and severe pneumonia; cerebral causes included various types of acute cerebrovascular diseases (cerebral infarction, intracerebral hemorrhage, and subarachnoid hemorrhage); metabolic causes included both acute and chronic renal failure; and septic causes included infections in various parts (including those without definite lesions) and septic shock of unknown cause.

To avoid confusion in the image analysis, the following cases were excluded: (1) suicidal patient (drug intoxication or self-inflicted injury); (2) patients with a known traumatic cause for cardiac arrest and patients who had known intrathoracic pathologies, such as lung cancer, tension pneumothorax, pulmonary thromboembolism, and severe sequelae of previous infection [11]; (3) patients who had chronic congestive heart failure [17]; (4) out-of-hospital cardiac arrest patients or patients with unsatisfactory sonograms due to severe chest deformity or subcutaneous emphysema.

### 2.2. Lung Ultrasonography Examinations

Once the cardiac arrest patient achieved ROSC after resuscitation, lung ultrasound examinations were performed by two experienced and qualified ICU doctors who were well trained by the Chinese Critical Ultrasound Study Group. Lung ultrasonography was performed by a MicroMaxx (Sonosite Inc., Bothell, WA, US) or Mindray M7 with a microconvex5 MHz, 9-cm-long probe. To perform lung ultrasound examination and illustrate the signs correctly, two experienced researchers who were blinded to the clinical data and other radiological features were responsible for performing lung ultrasound examination and image analysis. The two researchers, respectively, evaluated the images and then took the average value of the two scores as the LUS of the patient. The 12 lung regions were determined using the anatomic landmarks (apex, midaxillary line, external limit of the rib cage, mediastinum border, and diaphragm) [18]. Six standard areas are considered in each hemithorax as follows: anterior, lateral, and posterior regions; each area is divided into both upper and lower fields.

### 2.3. Assessment of Lung Injury

**Figure 1:**
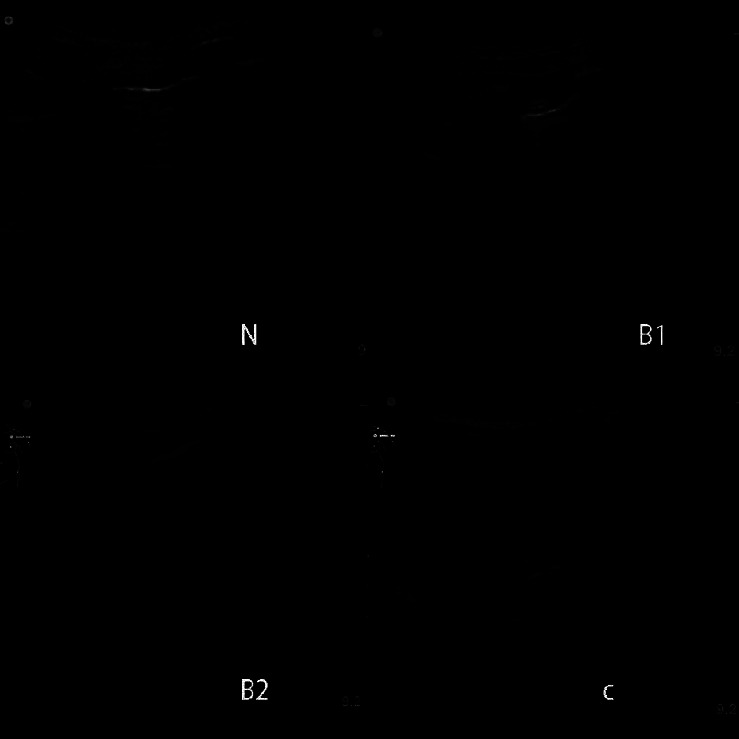
Different lung ultrasound aeration patterns. *Note*. Normal (N, score = 0), moderate (B1, score = 1), severe (B2, score = 2), and consolidation (C, score = 3).

The LUS protocol consisted of 12 scanning zones. The LUS was calculated according to B line, lung consolidation sign, and pleural sliding sign. We defined four ultrasound aeration patterns as follows [12, 19]: (1) normal sign: pleural sliding combined with A lines or less than two isolated B lines (N, score = 0); (2) moderate loss of lung aeration: multiple, clearly defined B lines (B1, score = 1); (3) severe loss of lung aeration: multiple coalescent B lines (B2, score = 2); and (4) lung consolidation (C, score = 3); they showed shred sign and tissue-like sign on LUS, which did not change during the respiratory cycle. We recorded the worst visible pattern for each region and calculated the sum of the scores as the LUS to assess the severity. LUS ranged, therefore, from 0 (all areas normally aerated) to 36 (all regions consolidated) [20]. Representative lung ultrasound images of patients with different LUSs are shown in Figure 1.

### 2.4. Statistical Analysis

Statistical analysis was done using SPSS20.0. Continuous variables are shown as the mean ± standard deviation (SD). Analysis of variance (ANOVA) was used to compare the continuous variables for normally distributed data. The Kruskal–Wallis test was used to analyze non-normally distributed data. Categorical variables were compared using the chi-square test or Fisher's exact test. We calculated the Pearson correlation coefficient to analyze whether two continuous variables were correlated. To assess the association of different variables with the cardiac arrest outcomes, univariate logistic regression was used. Univariate analysis was performed for all potential predictors of poor prognosis. Variables which were predicting the study outcomes in the univariate analysis with a *P* ≤ 0.10 were utilized in multivariate logistic regression to find out the independent predictors. For multivariate Cox regression models, clinical variables along with LUS were used to identify the independent risk factors of the 72 h survival time. A two-sided value of *P* < 0.05 was considered significant.

## 3. Results

### 3.1. General Characteristics

**Figure 2:**
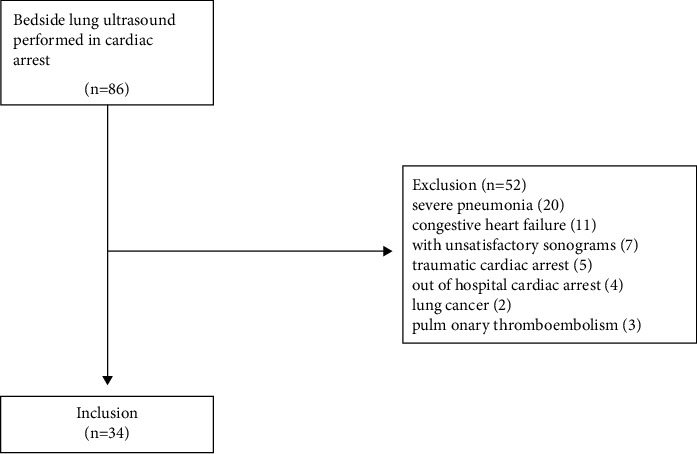
Flow diagram of patients' screening.

**Figure 3:**
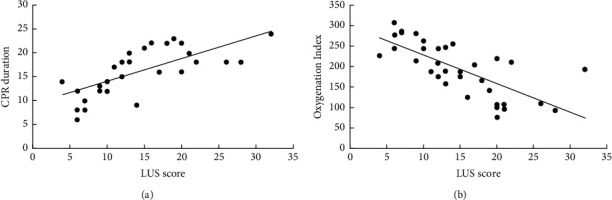
(a) Correlation plots among LUS, CPR, and OI. Correlation between the LUS and the CPR duration and (b) correlation between the LUS and the OI. *Note*. LUS: lung ultrasound score, CPR: cardiopulmonary resuscitation, and OI: oxygenation index.

A total of 86 cardiac arrest patients were screened, and only 34 were eventually included in the study (Figure 2). There were 10 (29.4%), 14 (41.2%), and 10 (29.4%) patients in the low, medium, and high LUS groups, respectively. The baseline clinical characteristics of patients according to different LUS levels (low, medium, and high) are summarized in Table 1. Compared with the lowest LUS group, the moderate group had a lower oxygenation index (OI) and the highest LUS group had the lowest oxygenation index (*P* < 0.001), longest duration of CPR (*P* < 0.001), and the lowest 72 h survival rate (*P* < 0.001). Different lung ultrasound aeration patterns of normal, moderate, severe, and consolidation are shown in Figure 1. Among the different groups, there were no significant differences in age, gender, and BMI (all *P* > 0.05). Significant positive linear correlations were found between LUS and CPR duration (*r* = 0.684; *P* < 0.001) while negative between LUS and OI (*r* = −0.718; *P* < 0.001) (Figure 3).

In the univariate analysis, the secondary outcomes of CPR were predicted to be significant by longer duration of CPR, lower OI, and presence of initial higher bedside LUS. Only initial higher LUS (adjusted odds ratio (aOR): 1.353, 95% confidence interval (CI): 1.018–1.797, and *P* = 0.037) independently predicted the secondary outcomes of CPR (Table 2). Similarly, the ROSC was predicted to be significant by lesser duration of CPR, higher OI, and presence of initial lower LUS in the univariate analysis. Just the adverse event (aOR: 0.04, 95% CI: 0.002–0.901, and *P* = 0.044) independently predicted the ROSC (Table 3). Variables which predicted the study outcomes in the univariate analysis with a *P* ≤ 0.10 were utilized in multivariate logistic regression to find out the independent predictors.

### 3.2. Predictors of the 72 h Survival Rate of Patients with CPR

Univariate Cox regression analysis revealed that LUS (aOR: 1.230, 95% CI: 1.133–1.335, and *P* < 0.001), CPR duration (aOR: 1.276, 95% CI: 1.121–1.453, and *P* ≤ 0.001), OI (aOR: 0.988, 95% CI: 0.018–0.995, and *P* < 0.001), secondary adverse events (aOR: 0.059, 95% CI: 0.017–0.194, and *P* = 0.001), and ROSC (aOR: 0.001, 95% CI: 0.001–0.965, and *P* = 0.001) were all predictors of 72 h survival time (Table 4). However, multivariate Cox regression analysis revealed that only LUS (aOR: 1.145, 95% CI: 1.014–1.294, and *P* = 0.029) and the secondary outcomes of CPR (aOR: 0.04, 95% CI: 0.002–0.921, and *P* = 0.002) were the independent predictors of 72 h survival rate of patients with CPR (Table 4).

## 4. Discussion

Postresuscitation lung injury is a common complication after cardiopulmonary resuscitation, and the mechanisms are extremely intricate. The lung ultrasound score (LUS) has been certified to predict disease outcomes and evolution of interstitial pneumonia in intensive care patients over time [21, 22]. It helps to guide the optimal setting of ventilator parameters and the process of withdrawal, guide volume management [23], and measure the severity of lung disease [24]. A study reported that there is a high correlation between the lung ultrasound score and the extravascular pulmonary water index and early lung ultrasound assessment can predict the occurrence of acute respiratory distress syndrome [25]. Picano and Pellikka [26] also suggested assessing pulmonary edema quantitativly or semiquantitativly through the number of B lines. Chiumello et al. [27] performed lung ultrasound examination on patients with acute respiratory distress and compared the results with CT results and found that the LUS was in good agreement with CT results, which could effectively evaluate pulmonary ventilation. At present, studies on lung injury after CPR are based on X-ray and CT, but few studies have reported on lung ultrasound.

This study aimed to investigate its clinical value in patients who had undergone cardiopulmonary resuscitation. We observed that patients with higher baseline LUSs in the study were more likely to have a lower OI, longer CPR duration, and a lower 72 h survival rate. We confirmed a negative correlation between the LUS and the PaO_2_/FiO_2_ (*r* = −0.718; *P* < 0.001) and a significant positive linear correlation between LUS and CPR duration (*r* = 0.684; *P* < 0.001).

In our study, the CPR duration (aOR: 1.676, 95% CI: 0.945–2.974, and *P* = 0.077) was not an independent predictor of ROSC, which contradicts the results of an earlier study reported [28]. Bhoi et al. [28] had included both out-of-hospital cardiac arrest (OHCA) and in-hospital cardiac arrest (IHCA) patients, whereas our study only included IHCA patients. The secondary outcomes of CPR occurred in 12 patients secondary to CPR. We found that the secondary outcomes of the CPR group had a higher LUS (19.92 ± 6.46) than without the secondary outcomes group (12.05 ± 5.16; *P* < 0.001). The LUS had a good clinical value for predicting secondary outcomes of CPR (aOR: 1.353, 95% CI: 1.018–1.797, and *P* = 0.037).

The LUS as a continuous variable was found to have a good predictive value for 72 h survival time of patients with CPR (hazard ratio: 1.145, 95% CI: 1.014–1.294, and *P* = 0.029) in the Cox models. Stratified by the level of the LUS, moderate LUS was strongly relevant to the 72 h survival rate as compared to the highest LUS (HR: 35.617, 95% CI: 1.520–83.460, and *P* = 0.026 vs. HR: 2.239, 95% CI: 0.304–16.492, and *P* = 0.429). The reason for this phenomenon is considered to be related to discharge in the highest LUS group, leading to reduced death.

Current research suggests that lung ultrasonography is readily available and is considered to be almost as accurate as computed tomography [29, 30]. The sum of the scores for all areas of the lung contusion (ALCs) was calculated on chest CT and defined as the lung contusion score (LCS). Jang et al. [31] reported that there is no correlation between the LCS and the duration of CPR (*r* = 0.285; *P* = 0.097). Jang et al. [31] also deemed that the LCS did not correlate with CPR duration. However, in our study, we quantified the LUS and found that CPR duration is an important factor related to lung injuries (*r* = 0.684; *P* = 0.001). Therefore, LUS may not be concordant with LCS. There are two possible reasons for this. First, solitary segmental or lobar distributed air space consolidation, defined as nonpulmonary contusion on CT image, were excluded in the LCS, while they were considered as consolidation on LUS and included in the LUS. Second, even if the study had ruled out patients with underlying lung disease, it might still have included some patients with developed lung disease who had not been diagnosed at time of admission.

Patients in our study were grouped according to the oxygenation index to assess the severity of lung injury as follows: mild group (200 mmHg < OI ≤ 300 mmHg), moderate group (100 mmHg < OI ≤ 200 mmHg), and severe group (OI ≤ 100 mmHg). Compared with the mild group, LUS in the moderate group and severe group was increased (*P* < 0.05) and the number of B line was increased in the 2 groups (*P* < 0.05). Compared with the moderate group, LUS and the number of B line in the severe group were equally increased (*P* < 0.05). Therefore, we confirmed that LUS can be used as an indicator to assess the lung injury related with CPR.

In this study, LUS was correlated with the CPR duration and OI, also a good predictor of secondary outcomes of CPR, and the 72-h survival rate after resuscitation. LUS could be an index to assess lung injury associated with CPR. Nevertheless, our findings need to be validated in future larger studies with stringent protocols to assess the clinical value of LUS.

### 4.1. Limitations

The study had some shortcomings. First, our study included a small number of patients, so the findings' generalizability is limited. Large-scale clinical studies are needed to confirm our results in the future. Along with that, a subgroup of patients was excluded due to anatomical difficulties associated with acquiring ultrasound images, which also limits the generalizability of the research. Furthermore, we have investigated the role of the “initial LUS” as a predictor of outcomes but the dynamic changes of lung injury were not considered in our study. After that, due to the fact of the retrospective nature, the duration of the study included the pandemic era, the use of experienced LUS performers, etc., present findings need to be validated in well-designed, higher population, practiced performers, and prospective studies to further confirm clinical values of LUS in CPR patients.

## 5. Conclusion

In our study, initial LUS predicted the adverse events secondary to CPR and 72 h survival rate after resuscitation and the association was statistically significant. Further studies with a larger sample size, including patients with dynamic changes of lung injuries, and involving CT images should be conducted in the future to validate our results.

## Figures and Tables

**Table 1:** General characteristics of patients stratified by the level of the LUS.

Variables	Total population (*n*: 34)	LUS score			*P*
		Low ≤ 10 (*n*: 10)	Moderate 11–19 (*n*: 14)	High ≥ 20 (*n*: 10)	
Demographic data					
Age (year)	53.97 ± 11.23	53.90 ± 9.30	49.50 ± 12.40	60.30 ± 8.86	0.063
Male, *n* (%)	20 (58.8)	6 (60)	8 (57.1)	6 (60)	0.986
BMI (kg/m^2^)	24.59 ± 1.69	24.20 ± 1.74	24.60 ± 1.49	24.96 ± 1.98	0.621
Duration of CPR	16.32 ± 4.68	10.90 ± 2.77	18.43 ± 3.63	18.80 ± 2.70	<0.001
PaO_2_:FiO_2_ (mmHg)	194.21 ± 65.28	262.40 ± 29.52	190.50 ± 38.87	131.20 ± 54.52	<0.001
Causes of cardiac arrest, *n* (%)					
Respiratory	10 (29.4)	2 (20)	4 (28.6)	4 (40)	0.725
Cardiac problem	8 (23.5)	1 (10)	4 (28.6)	3 (30)	0.600
Sepsis	6 (17.6)	1 (10)	2 (14.3)	3 (30)	0.619
Metabolic causes	5 (14.7)	0 (0)	2 (14.3)	3 (30)	0.234
Brain problem	3 (8.8)	0 (0)	1 (15.8)	2 (20)	0.462
Drug overdose	2 (5.9)	1 (10)	0 (0)	1 (10)	0.501
72-hour survival rate	8 (23.5)	6 (60)	2 (14.3)	0 (0)	0.004

*Note*. BMI: body mass index, LUS: lung ultrasound score, and CPR: cardiopulmonary resuscitation.

**Table 2:** Predictors of the secondary outcomes of CPR.

Variables	Univariate analysis		Multivariate analysis	
	Unadjusted OR	*P*	Adjusted OR	*P*
Age	1.002 (0.941–1.068)	0.939		
Gender	0.969 (0.232–4.042)	0.966		
BMI	0.958 (0.628–1.462)	0.843		
LUS score	1.275 (1.072–1.516)	0.006	1.353 (1.018–1.797)	0.037
CPR duration	1.307 (1.042–1.639)	0.02	1.214 (0.882–1.670)	0.234
OI	0.987 (0.975–1.000)	0.044	1.014 (0.989–1.040)	0.271

*Note*. OR: odds ratio, BMI: body mass index, LUS: lung ultrasound score, CPR: cardiopulmonary resuscitation, and OI: oxygenation index.

**Table 3:** Predictors of return of spontaneous circulation.

Variables	Univariate analysis		Multivariate analysis	
	Unadjusted OR	*P*	Adjusted OR	*P*
Age	1.002 (0.942–1.067)	0.939		
Gender (man)	2.045 (0.477–8.774)	0.335		
BMI	1.200 (0.781–1.842)	0.405		
LUS score	1.373 (1.112–1.696)	0.003	1.131 (0.735–1.739)	0.575
CPR duration	1.809 (1.195–2.739)	0.005	1.676 (0.945–2.974)	0.077
OI	0.977 (0.962–0.993)	0.004	0.994 (0.957–1.033)	0.755
Adverse event	0.032 (0.005–0.221)	0.001	0.040 (0.002–0.921)	0.044

*Note*. OR: odds ratio, BMI: body mass index, LUS: lung ultrasound score, CPR: cardiopulmonary resuscitation, and OI: oxygenation index.

**Table 4:** Predictors of 72 h survival by the Cox proportional hazard model.

Variables	Univariate analysis		Multivariate analysis	
	Unadjusted OR	*P*	Adjusted OR	*P*
Age	1.009 (0.977–1.041)	0.600		
Gender (man)	0.989 (0.453–2.159)	0.978		
BMI	1.096 (0.877–1.370)	0.419		
LUS score	1.230 (1.133–1.335)	0.001	1.145 (1.014–1.294)	0.029
CPR duration	1.276 (1.121–1.453)	0.001	1.094 (0.925–1.295)	0.294
OI	0.988 (0.982–0.995)	0.001	1.002 (0.992–1.012)	0.648
Adverse event	0.059 (0.018–0.194)	0.001	0.040 (0.002–0.921)	0.002
ROSC	0.001 (0.001–0.965)	0.049	0.001 (0.001–40.05)	0.906

*Note*. OR: odds ratio, BMI: body mass index, LUS: lung ultrasound score, CPR: cardiopulmonary resuscitation, OI: oxygenation index, and ROSC: return of spontaneous circulation.

## Data Availability

The datasets used to support the findings of this study are available on request from the corresponding author.
